# Assessment of Allergen-Responsive Regulatory T Cells in Experimental Asthma Induced in Different Mouse Strains

**DOI:** 10.1155/2021/7584483

**Published:** 2021-12-10

**Authors:** C. T. Azevedo, A. C. Cotias, A. C. S. Arantes, T. P. T. Ferreira, M. A. Martins, P. C. Olsen

**Affiliations:** ^1^Laboratório de Inflamação, Instituto Oswaldo Cruz, FIOCRUZ, Brazil; ^2^Laboratório de Estudos em Imunologia, Departamento de Análises Clínicas e Toxicológicas, Faculdade de Farmácia, Universidade Federal do Rio de Janeiro (UFRJ), Av. Carlos Chagas Filho 373, Bloco A 2º Andar sala 07, Cidade Universitária, Ilha do Fundão, CEP. 21941-902 Rio de Janeiro, Brazil

## Abstract

**Background:**

Regulatory T cells (Tregs) are important in regulating responses to innocuous antigens, such as allergens, by controlling the Th2 response, a mechanism that appears to be compromised in atopic asthmatic individuals. Different isogenic mouse strains also have distinct immunological responses and susceptibility to the experimental protocols used to develop lung allergic inflammation. In this work, we investigated the differences in the frequency of Treg cell subtypes among A/J, BALB/c, and C57BL/6, under normal conditions and following induction of allergic asthma with ovalbumin (OVA).

**Methods:**

Subcutaneous sensitization followed by 4 consecutive intranasal OVA challenges induced asthma characteristic changes such as airway hyperreactivity, inflammation, and production of Th2 cytokines (IL-4, IL-13, IL-5, and IL-33) in the lungs of only A/J and BALB/c but not C57BL/6 strain and evaluated by invasive whole-body plethysmography, flow cytometry, and ELISA, respectively.

**Results:**

A/J strain naturally showed a higher frequency of CD4^+^IL-10^+^ T cells in the lungs of naïve mice compared to the other strains, accompanied by higher frequencies of CD4^+^IL-4^+^ T cells. C57BL/6 mice did not develop lung inflammation and presented higher frequency of CD4^+^CD25^+^Foxp3^+^ Treg cells in the bronchoalveolar lavage fluid (BALF) after the allergen challenge. In *in vitro* settings, allergen-specific stimulation of mediastinal LN (mLN) cells from OVA-challenged animals induced higher frequency of CD4^+^IL-10^+^ Treg cells from A/J strain and CD4^+^CD25^+^Foxp3^+^ from C57BL/6.

**Conclusions:**

The observed differences in the frequencies of Treg cell subtypes associated with the susceptibility of the animals to experimental asthma suggest that CD4^+^CD25^+^Foxp3^+^ and IL-10-producing CD4^+^ Treg cells may play different roles in asthma control. Similar to asthmatic individuals, the lack of an efficient regulatory response and susceptibility to the development of experimental asthma in A/J mice further suggests that this strain could be preferably chosen in experimental models of allergic asthma.

## 1. Introduction

Asthma is a chronic inflammatory disease of the lungs which main symptoms are airway obstruction and reversible airway hyperresponsiveness (AHR) to nonspecific irritants. Several risk factors are involved with the development of this complex heterogeneous disease such as genetic predisposition and environmental exposure, leading to distinct phenotypes. Allergic asthma is the most common and easily treated phenotype, which is generally associated with early onset and a Th2 immune response driven to inhaled allergens, leading to high serum IgE levels and eosinophilic lung infiltration. Regulatory T (Treg) cells have a key role in the maintenance of tolerance toward allergens in lung mucosa through inhibiting the activity of effector inflammatory cells that orchestrate the asthma allergic response [1–3]. These cells were initially described as a population of CD4 lymphocytes that expresses CD25 (IL-2 receptor *α* chain) [4, 5], and later, it was identified that Foxp3 (Forkhead Box 3) is a specific Treg cell marker essential to their function [6, 7]. Different populations of Tregs have been described with distinct origins, mechanisms of suppression, and antigenic targets [8]. Tregs can suppress the immune response by directly inhibiting activation of Th2 and Th17 cells and activity of eosinophils and basophils through IL-10 [9–11], as well as mast cell degranulation through cell-to-cell contact [12].

The role of Tregs in asthmatic patients has been much debated; adult asthmatic patients with either stable or severe symptoms present lower amounts of Treg cells in the airways and blood, and their Tregs also present reduced suppressor activity [13, 14]. In pediatric asthmatic patients, the percentage of Tregs in the airways was also found to be reduced [15]. Controversially, other studies have shown that the number of lung regulatory lymphocytes was increased in moderate to severe adult asthmatic patients compared with mild asthmatics and healthy subjects [16], especially following inhaled allergen provocation [17]. Discrepancies in these studies might involve different methods for analyzing Tregs and diverse cohorts. However, it is a consensus that there is a functional impairment of Tregs in asthmatics, especially concerning their ability to control Th2 responses [18]. Nevertheless, Tregs expressing Foxp3 or IL-10 correlate with decreased allergic lung inflammation and accumulate due to specific allergy immunotherapy [3, 19–21].

It is unclear also in murine models of asthma what are the roles of distinct Treg subpopulations. Divergence in studies can be a result of different antigen, mouse strain, and experimental protocols used. Therefore, we seek here to study the differences in the frequency of Foxp3 and IL-10 producing Treg cells between A/J, BALB/c, and C57BL/6 mice that have different susceptibility to develop allergic lung inflammation, using an allergy asthma model induced with ovalbumin.

## 2. Methods

### 2.1. Mice

Experiments were conducted with 2 months old (18-20 g) male and female A/J, BALB/c, and C57BL/6 wild-type mice. All mice were obtained at the Oswaldo Cruz Foundation animal facility ICTB/CECAL/FIOCRUZ (Rio de Janeiro, Brazil) and were kept at the Ozório de Almeida Building/FIOCRUZ animal-housing facility with controlled room temperature (22–25°C) and a 12 h light–dark cycle. Procedures involving the care and use of mice were examined and approved by the Animal Ethics Committee of the Oswaldo Cruz Foundation (CEUA-FIOCRUZ), Rio de Janeiro, Brazil, on August 25th of 2015 under the approval code L-030/15, for A/J, BALB/c, and C57BL/6 mice.

### 2.2. Ovalbumin (OVA) Sensitization and Challenge Protocols

The experimental asthma model was performed as described previously in detail [22], with the following modifications. Intranasal OVA challenges were given once a day from day 19 to 22 postsensitization (day 0).

### 2.3. Invasive Assessment of Lung Function

Mouse airway hyperresponsiveness was assessed 24 h after the final antigen challenge, as previously described [23], with the exception that the increasing concentrations of methacholine aerosolized were 0, 3, 9, 27, and 81 mg/mL.

### 2.4. Histological Analysis of Lung Eosinophilic Infiltration

Mice were killed with sodium pentobarbital (500 mg/kg i.p., (THIOPENTAX®, Cristália)) after the lung function assessment. The lungs were fixed, embedded in Paraplast (Sigma), prepared for histological sections of 4 *μ*m, and stained with Llewellyn's Sirius Red (Direct Red 80, C.I 35780; Sigma). Eosinophilic infiltration was evaluated around the airways, bronchial epithelium, and adventitia through an integrating eyepiece (10,000 *μ*m^2^ of total area) at a microscopic magnification of 1000x. Eosinophil counts were performed in 24 randomly selected fields/lung of each mouse and expressed as the average of eosinophils/10^4^ (*μ*m^2^) [22, 24].

### 2.5. Cell Recovery from the Airway Lumen, Lung Tissue, Thyme, and Lymph Nodes

For airway lumen, mouse tracheas were cannulated, and bronchoalveolar lavage fluid (BALF) was obtained by gentle aspiration of 0.5 mL PBS (1x, (Sigma)), repeated three times. BALF was centrifuged at 250 g for 10 minutes at 4°C, and cell pellets were resuspended in 1x PBS.

For lung tissue, one lung lobe was mechanically chopped and incubated at 37°C for 1 h in RPMI-1640 (Sigma) containing 10% fetal calf serum (FCS, (Gibco)), plus 0.15 mg/mL collagenase (type 1, Gibco) and 25 *μ*g/mL DNAse (type 1, Roche Diagnostics). Cells were recovered by filtration through a 40 *μ*m cell strainer, washed 3 times, and resuspended in 5 mL RPMI +10% FCS.

For thyme and lymph nodes, mediastinal lymph nodes (mLN), or total lymph nodes (cervical, axial, and inguinal) were carefully extracted, placed into 1 mL of 1x PBS, and homogenized in Potter-Elvehjem (Sigma). Lymph node cells retrieved for cell culture were recovered by filtration through a 40 *μ*m cell strainer, washed once, and resuspended in DMEM (Gibco) +20% FCS.

Total leucocytes from the BALF, lung digested tissue, thyme, and lymph nodes were counted in a Neubauer chamber after dilution using Turk's solution (2% acetic acid).

### 2.6. Detection of Cytokines

Cytokine levels were detected in lung tissue, thymus, or lymph node homogenates. Briefly, tissues were homogenized in the presence of a lysis buffer solution (1x PBS, 0.1% Triton X-100, and a complete™ Protease Inhibitor Cocktail tablet (Roche Diagnostics)) and centrifuged (3500 rpm for 10 min at 4°C), and the resulting supernatant was assayed. Cytokine was also detected from the supernatant of *in vitro* T cell assays.

Sandwich ELISAs for IL-5, IL-10, IL-17, IL-33, and TGF-*β* were performed as per the manufacturer's instructions (R&D Systems), as were assayed for IL-4 and IL-13 (Invitrogen™ Novex).

### 2.7. *In Vitro* T Cell Polarization

Pooled cervical, axial, and inguinal lymph node cells (10^6^/well) from *naïve* A/J, BALB/c, and C57BL/6 mice were extracted. Cells obtained were exposed to IL-2 (100 UI/mL, Peprotech) alone or with the addition of plate-immobilized anti-CD3e (2 *μ*g/mL, BD), anti-CD28 (2 *μ*g/mL, BD), and TGF-*β* (2 ng/mL, Peprotech) for 72 h at 37°C and 5% CO_2_ [25]. Cells were retrieved for immunophenotyping, and when relevant, supernatant was evaluated for detection of cytokine secretion, as described on the previous section.

mLN cells from OVA-challenged mice were exposed *in vitro* to IL-2 (100 UI/mL) alone or with the addition of OVA (0.5 mg/mL) with or without TGF-*β* (2 ng/mL) for 72 h at 37°C and 5% CO_2_.

### 2.8. Flow Cytometry Immunophenotyping

Cells were blocked with normal sheep serum and stained with monoclonal anti-mouse CD4 antibodies (RM4-5 clone; eBiosciences; San Diego, CA, USA). Intracellular staining of CD4 T cells was performed using anti-mouse IL-10-PE antibody (JES5-16E3 clone), anti-IL-4 PECy7 (11B11 clone), and anti-IL-17 eFluor 660 (eBio17B7 clone) or the respective controls IgG2b*κ* PE, IgG1*κ* PECy7, and IgG2a*κ* eFluor 660 (all against mouse proteins, from eBioscience). Treg cells were characterized by staining for CD4, CD25, and Foxp3, according to the manufacturer's instructions (Mouse Treg Staining Kit, eBiosciences).

Pulmonary myeloid dendritic cells (CD11c^+^CD11b^+^Gr1^−^B220^−^) were characterized as previously [26], using anti-Gr1 FITC (RB6-8C5 clone) anti-CD11c PE (N418 clone), anti-CD11b PEcy7 (M1/70 clone), and anti-B220 APC (RA3-6B2 clone) or the respective controls IgG2b*κ* FITC, IgG armenian hamster PE, IgG2b*κ* PECy7, and IgG2a*κ* APC (all against mouse proteins, from eBiosciences).

All data were acquired in a FACSCalibur flow cytometer (BD Biosciences Immunocytometry Systems, San Jose, CA, USA) and analyzed using the FlowJo X 10 and 7.6.5 software (Tree Star Inc., Ashland, OR, USA). Percentages of total cells were presented in bar graphs.

### 2.9. Statistical Analysis

Data were presented as mean standard deviation or median (interquartile range) for a group of 3-10 animals. Statistical analysis was performed using a *t*-test or one-way analysis of variance (ANOVA) followed by Newman-Keuls test. Lung function data were analyzed using two-way ANOVA followed by Bonferroni correction. All tests were performed in GraphPad Prism 6.00 (GraphPad Software, La Jolla, CA, USA). *P* values ≤ 0.05 were considered statistically significant.

## 3. Results

**Figure 1:**
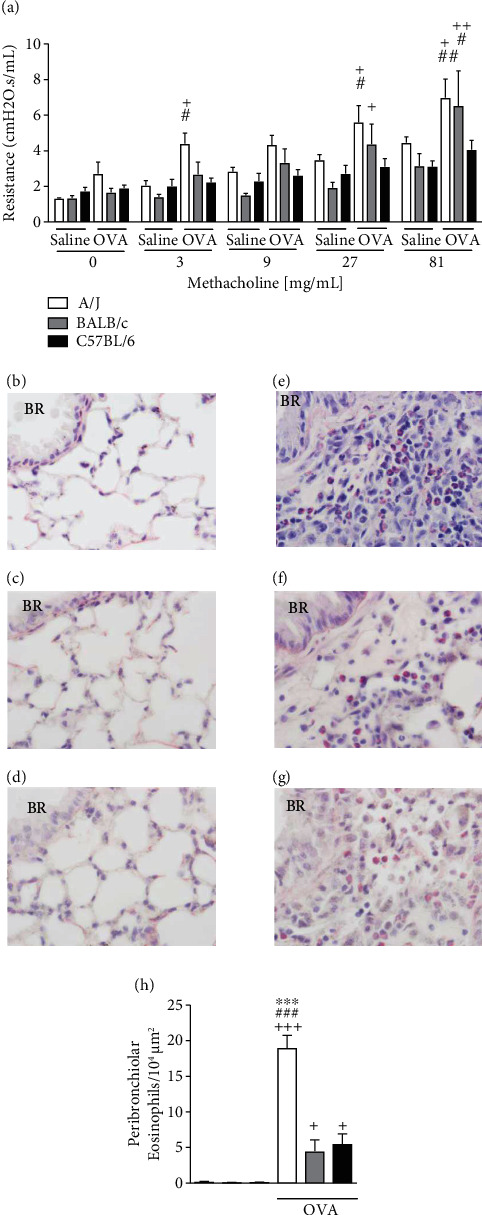
Assessment of airway resistance and eosinophilic inflammation in saline or OVA-challenged A/J, BALB/c, or C57BL/6 mice. Lung resistance was induced by increasing concentrations of methacholine bronchoprovocation, evaluated 24 h after the last OVA challenge (a). Photomicrographs of representative airways stained by Sirius Red of saline-challenged A/J (b), BALB/c (c), or C57BL/6 (d) and OVA-challenged A/J (e), BALB/c (f), or C57BL/6 (g) mice (original magnification 1000x). Quantitative data for eosinophils is shown in (h). Values represent the mean ± SEM of 4-6 animals per group. ^+^*P* < 0.05, ^++^*P* < 0.01, and ^+++^*P* < 0.001 as compared to the respective saline-challenged mouse strain group. ^#^*P* < 0.05, ^##^*P* < 0.01, and ^###^*P* < 0.001 as compared to OVA-challenged C57BL/6 mice. ^∗∗∗^*P* < 0.001 as compared to OVA-challenged BALB/c mice.

Lung resistance was evaluated through whole-body plethysmography where mice were subjected to increasing concentrations of nebulized methacholine, assessed after 4 sequential daily challenges of either OVA or saline. As expected, genetically hyperresponsive A/J mice [27–31] when challenged with OVA showed increased airway resistance compared to the saline-challenged A/J mice already with 3 mg/mL methacholine bronchoprovocation (Figure 1(a)). The increase in airway resistance compared to the same mouse strain challenged with saline was observed in BALB/c mice after the 27 mg/mL methacholine bronchoprovocation but not in C57BL/6 mice (Figure 1(a)). Analysis of the airway resistance data showed that saline-challenged A/J mice presented higher area under the curve (11.3 arbitrary units (AU)), compared to saline-challenged BALB/c (7.1 AU) and C57BL/6 (9.5 AU) mice, as well as when compared to OVA-challenged C57BL/6 mice (10.9 AU), but not to OVA-challenged BALB/c (14.5 AU) or A/J (19.2 AU) mice. Eosinophilic lung infiltration was induced by OVA-challenge in all strains, although it was significantly more pronounced in A/J OVA-challenged mice (Figures 1(b)–1(h)).

**Figure 2:**
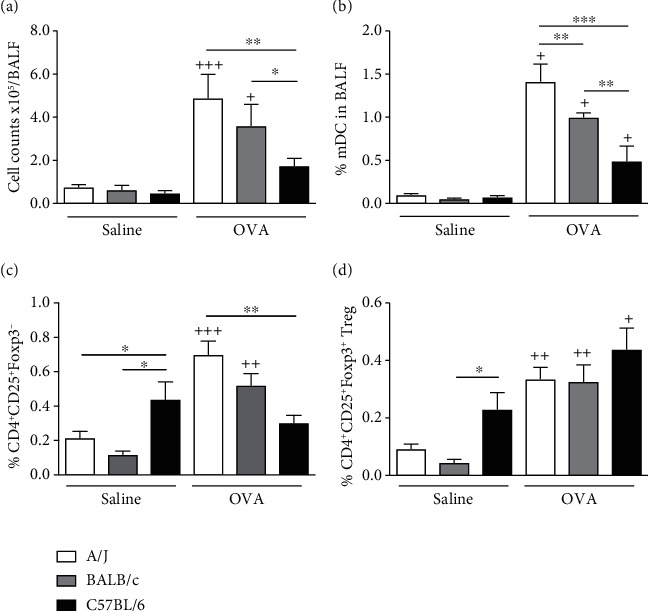
Characterization of inflammatory and regulatory cells in the airways of A/J, BALB/c, and C57BL/6 mice challenged with either OVA or saline. Total leukocyte numbers (a), frequency of pulmonary mDCs (b), CD4^+^CD25^+^Foxp3^−^ (c), and CD4^+^CD25^+^Foxp3^+^ Treg (d) cells in the BALF were evaluated. Values represent mean ± SEM 4-6 animals per group. ^+^*P* < 0.05, ^++^*P* < 0.01, or ^+++^*P* < 0.001 as compared to saline-challenged group. ^∗^*P* < 0.05, ^∗∗^*P* < 0.01, and ^∗∗∗^*P* < 0.001 are comparisons of different OVA-challenged mouse strains.

After 4 allergenic provocations, OVA-challenged A/J mice presented higher cell counts and percentage of myeloid dendritic cells (mDC) and activated T cells in the bronchoalveolar lavage fluid (BALF) when compared to A/J saline-challenged (Figures 2(a)–2(c)). The same OVA challenge protocol induced an increase in cell counts, percentage of mDC, and activated T cells in the airway lumen of BALB/c when compared to their saline-challenge counterpart, which was not observed in C57BL/6 mice (Figures 2(a)–2(c)). Actually, C57BL/6 saline-challenged mice presented more activated T cells than the other saline-challenged strains, but this percentage was not increased by the allergen challenge (Figure 2(c)). Interestingly, saline-challenged C57BL/6 mice were also the ones to present increased percentage of CD4^+^CD25^+^Foxp3^+^ regulatory T cells in the airway lumen (Figure 2(d)). OVA challenge induced an increase in the percentage of these Tregs in the airway lumen of the 3 strains studied (Figure 2(d)).

**Figure 3:**
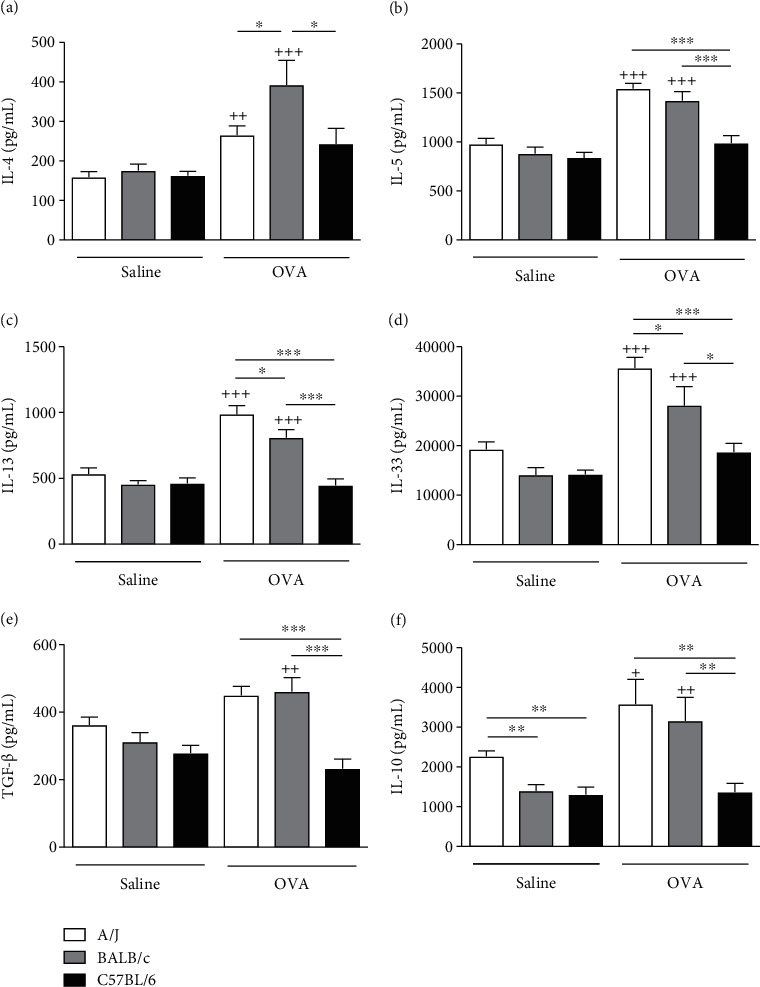
Production of Th2 and anti-inflammatory cytokines in the lung tissue of A/J, BALB/c, and C57BL/6 mice challenged with either OVA or saline. Detection of cytokines IL-4 (a), IL-5 (b), IL-13 (c), IL-33 (d), TGF-*β*(e), and IL-10 (f) in the lung homogenate by ELISA. Values represent mean ± SEM of 5-6 animals per group. ^+^*P* < 0.05 as compared to saline-challenged group. ^++^*P* < 0.01 and ^+++^*P* < 0.001 as compared to saline-challenged group. ^∗^*P* < 0.05, ^∗∗^*P* < 0.01, and ^∗∗∗^*P* < 0.001 are comparisons of different OVA-challenged mouse strains.

OVA challenge induced an increase in secretion of Th2 cytokines, such as IL-4, IL-5, IL-13, and IL-33 in the lungs of A/J and BALB/c mice but not in C57BL/6 (Figures 3(a)–3(d)). The increase in IL-13 and IL-33 secretion was more prominent in the lungs of A/J OVA-challenged mice than in BALB/c, whereas BALB/c was the one to secrete more IL-4 post-OVA challenge. TGF-*β* secretion was significantly enhanced by OVA challenge in BALB/c mice but not in C57BL/6 (Figure 3(e)). Although A/J OVA-challenged mice did not present statistically significant increase in lung TGF-*β* levels than the saline-challenged A/J, it was higher than levels produced by OVA-challenged C57BL/6 mice (Figure 3(e)). OVA challenge also induced lung secretion of IL-10 in A/J and BALB/c but not in C57BL/6 mice (Figure 3(f)). Interestingly, A/J saline-challenged mice already presented more IL-10 lung secretion than the other strains (Figure 3(f)). IL-17 lung production was not different in the 3 mouse strains analyzed, independent of the lung challenge used (data not shown).

**Figure 4:**
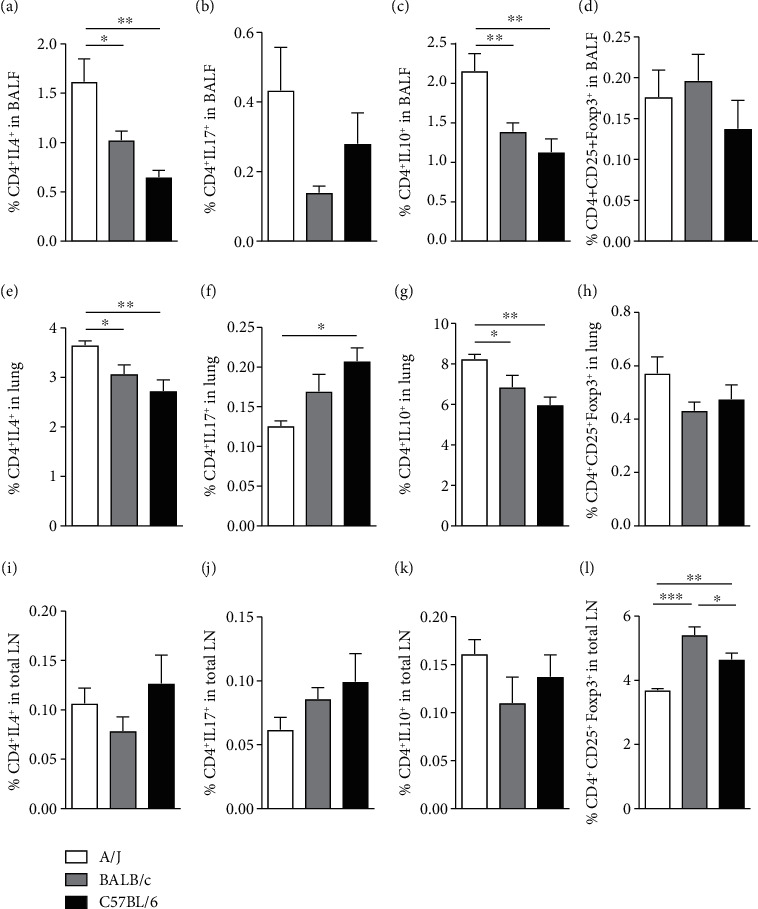
Frequency of Th2, Th17, and regulatory T cells in the airways, lung parenchyma, and lymph nodes of A/J, BALB/c, and C57BL/6 naïve animals. Flow cytometric analysis of CD4^+^ cells producing IL-4, IL-17, and IL-10, and CD4^+^CD25^+^Foxp3^+^ Tregs, respectively, in the BALF (a–d), lung tissue (e–h), and in total lymph nodes (i–l). Values represent mean ± SEM of 4-5 animals per group. ^∗^*P* < 0.05, ^∗∗^*P* < 0.01, ^∗∗∗^*P* < 0.001.

We investigated the percentage of different subpopulations of CD4 T cells in naïve mice of the 3 strains in different compartments. Naïve A/J mice showed higher percentage of CD4^+^IL-4^+^ and CD4^+^IL10^+^ T cells, compared to the other strains, in the BALF (Figures 4(a) and 4(c)) and in the lungs (Figures 4(e) and 4(g)). There was no significant difference in the percentage of CD4^+^IL17^+^ and CD4^+^CD25^+^Foxp3^+^ cells in the BALF of the 3 strains (Figures 4(b) and 4(d)). In the digested lungs of C57BL/6 mice, there was an increased percentage of CD4^+^IL17^+^ cells, compared to A/J, but again, there was no difference in CD4^+^CD25^+^Foxp3^+^ cells among the strains (Figures 4(f) and 4(h)). There was no difference in CD4^+^IL-4^+^, CD4^+^IL17^+^, and CD4^+^IL10^+^ T cells in total lymph nodes of the 3 strains (Figures 4(i), 4(j), and 4(l)), but A/J presented less CD4^+^CD25^+^Foxp3^+^ cells than the other strains in these compartments (Figure 4(m)). Production of TGF-*β* is higher than production of IL-10 in total LNs and thyme of 3 strains of naïve mice analyzed (data not shown). There was no particular strain of naïve mice presenting difference in the production of IL-10 and TGF-*β* in total LNs and thyme (data not shown).

**Figure 5:**
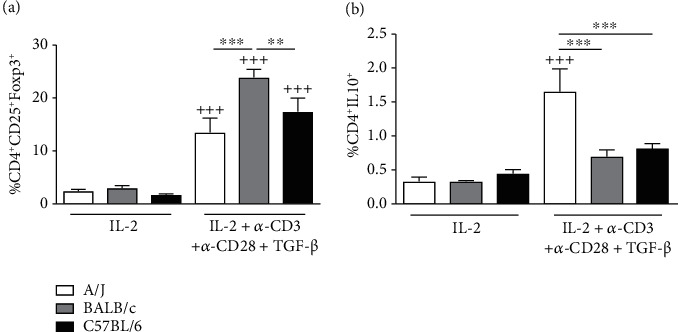
*In vitro* expansion of CD4^+^CD25^+^Foxp3^+^ and CD4^+^IL10^+^ cell populations from lymph nodes of A/J, BALB/c, or C57BL/6 naive mice. Cells from cervical, axillary, and inguinal lymph nodes were cultured for 72 h in the presence of IL-2 or IL-2 + TGF-*β* + anti-CD3 + anti-CD28, and subpopulations of CD4^+^CD25^+^Foxp3^+^ (a) or CD4^+^IL-10^+^ (b) were analyzed by flow cytometry. Values represent mean ± SEM of 4-5 animals per group. ^+++^*P* < 0.001 as compared to the same mouse strain cells that received only IL-2. ^∗∗^*P* < 0.01 and ^∗∗∗^*P* < 0.001 are comparisons of different mouse strains stimulated with IL-2 + TGF-*β* + anti-CD3 + anti-CD28.

To investigate the expansion of regulatory T cells in the 3 different strains, total lymph nodes retrieved from naïve mice were antigenically stimulated *in vitro* in the presence of TGF-*β*. Activation of T cells from naïve mice in the presence of TGF-*β* induced expansion of CD4^+^CD25^+^Foxp3^+^ T reg cell population in all strains but was significantly higher in cells from BALB/c mice (Figure 5(a)). The same protocol induced significant expansion of CD4^+^IL10^+^ Treg cells only in A/J mice (Figure 5(b)).

**Figure 6:**
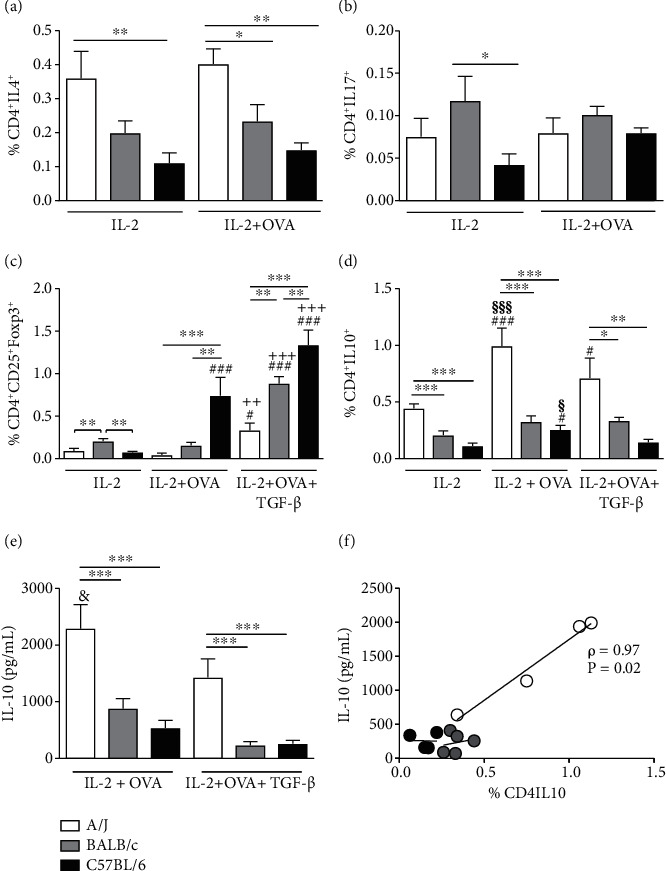
*In vitro* expansion of CD4^+^IL4^+^, CD4^+^IL17^+^, CD4^+^CD25^+^Foxp3^+^, and CD4^+^IL10^+^ cell populations from lymph nodes of A/J, BALB/c, or C57BL/6 OVA-challenged mice. Cells from mediastinal lymph nodes were cultured for 72 h in the presence IL-2, IL-2 + OVA, or IL-2 + OVA + TGF-*β*, and subpopulations of CD4^+^IL4^+^ (a) and CD4^+^IL17^+^ (b) CD4^+^CD25^+^Foxp3^+^ (c) or CD4^+^IL10^+^ (d) were analyzed by flow cytometry. IL-10 production in the supernatant of cultured cells was detected by ELISA (e). Values represent mean ± SEM of 4-5 animals per group. ^#^*P* < 0.05 and ^###^*P* < 0.001 as compared to the same mouse strain group that received only IL-2. ^++^*P* < 0.01 and ^+++^*P* < 0.001 as compared to the same mouse strain group that received IL-2 + OVA. ^§^*P* < 0.05 and ^§§§^*P* < 0.001 as compared to the same mouse strain group that received IL-2 + OVA + TGF-*β*. ^&^*P* < 0.05 as compared to the same strain between different stimulus. ^∗^*P* < 0.05, ^∗∗^*P* < 0.01, ^∗∗∗^*P* < 0.001. Correlation between frequency of CD4^+^IL10^+^ cells and IL-10 production in cultures stimulated with OVA and TGF-*β* (f). Pseudo-*r* = 0.97, *P* = 0.02 by univariate analysis.

Culture of cells from lung draining lymph nodes of OVA-challenged mice with IL-2 showed A/J mice presented higher percentage of CD4^+^IL4^+^ T cells, when compared to BALB/c and C57BL/6 mice (Figure 6(a)). The OVA *in vitro* stimulation of cells from lung draining lymph nodes of OVA-challenged mice did not change much the percentage of CD4^+^IL4^+^ T cells in the 3 different strains. Although the percentage of CD4^+^IL17^+^ T cells from OVA-challenged mouse lung draining lymph nodes maintained by IL-2 *in vitro* was higher in BALB/c mice, compared to C57BL/6, this difference reduced when cells were stimulated with OVA *in vitro* (Figure 6(b)). Culture of cells from lung draining lymph nodes of OVA-challenged mice with IL-2 showed BALB/c presented higher percentage of CD4^+^CD25^+^Foxp3^+^ Treg cells. After the *in vitro* antigenic stimulus with OVA, there was a very significant increase in the percentage of CD4^+^CD25^+^Foxp3^+^ Treg cells from C57BL/6 mouse cells when compared to A/J and BALB/c (Figure 6(c)). Addition of TGF-*β* with the OVA *in vitro* stimulus favored an increase in the percentage of CD4^+^CD25^+^Foxp3^+^ Treg cells from all strains analyzed, but their percentage kept higher when cells came from OVA activated C57BL/6 mice (Figure 6(c)). After *in vitro* IL-2 stimulation of OVA-challenged mouse lung draining lymph node cells, A/J mice presented higher percentage of CD4^+^IL10^+^ cells, compared to BALB/c and C57BL/6 mice (Figure 6(d)). OVA activation led to a further increase in the percentage of CD4^+^IL10^+^ T cells from A/J mice, which was maintained in the presence of TGF-*β* (Figure 6(d)). Accordingly, *in vitro* production of IL-10 from cells obtained from A/J OVA-challenged mice, either stimulated with OVA or OVA and TGF-*β*, was higher than in supernatants of cells obtained from BALB/c or C57BL/6 OVA-challenged mice (Figure 6(e)). The percentage of CD4^+^IL10^+^ T cells correlated positively with the production of IL-10 in cultures of A/J OVA-challenged mouse cells in the presence of IL-2, OVA, and TGF-*β* (Figure 6(f)). This correlation was not observed in cells obtained from BALB/c or C57BL/6 mice.

## 4. Discussion

This study is aimed at investigating the differences in frequencies of Treg cell subtypes among naïve A/J, BALB/c, and C57BL/6 mice and following allergenic stimulation with OVA *in vivo* and *in vitro*. Our results showed that C57BL/6 mice presented the highest lung levels of CD4^+^CD25^+^Foxp3^+^ cells in the presence or absence of allergen provocation and failed to develop asthma characteristic features following allergen provocation. Additionally, the *in vitro* OVA allergenic challenge led to a pronounced increase in the percentage of CD4^+^CD25^+^Foxp3^+^ cells from C57BL/6 mice, compared to the other strains. A/J and BALB/c mice developed characteristic lung inflammation and AHR with the OVA lung challenge, accompanied by a TGF-*β*-dependent increase in the levels of CD4^+^CD25^+^Foxp3^+^ cells *in vitro*. Naïve A/J mice presented higher percentage of CD4^+^IL10^+^ cells in the lungs, when compared to the other strains. Although OVA-challenged-A/J mice presented robust lung inflammation and AHR, OVA stimulation led to a further increase in the percentage of CD4^+^IL10^+^ cells and IL-10 production, independent of TGF-*β*. The lack of an efficient regulatory response as well as the higher propensity to develop severe asthma changes suggests that A/J mice might be a preferable model concerning the identification of novel therapeutic targets and the screening of compounds active in difficult-to-treat asthma.

Previous investigations have demonstrated exacerbated lung inflammation and AHR in OVA-challenged A/J mice, compared to BALB/c and C57BL/6 [32, 33]. Overall, our data corroborate these studies, since OVA-challenged A/J mice showed increased airway resistance and lung infiltration of leukocytes, including activated T cells and mDCs, subsets that play immunostimulatory roles in experimental asthma [34]. In addition, A/J mouse lung challenged with OVA secreted more IL-33 and IL-13, which are involved in the activation of ILC2 cells that induce lung eosinophil accumulation and AHR, and in goblet cell metaplasia and AHR, respectively. The hallmark Th2 immune response of allergic asthma characterized by increased secretion of IL-13 and IL-4 is directly associated with AHR, a fundamental aspect of disease symptoms and morbidity [30, 35]. Among the mouse strains evaluated, A/J naïve mice presented increased frequency of Th2 cells expressing IL-4 in the lungs and AHR, corroborating previous data showing A/J increased AHR even in the noninflammatory state [30]. After the OVA allergenic stimulus, frequency of CD4^+^IL-4^+^ cells was still higher in A/J mouse, compared to BALB/c and C57BL/6. Nonetheless, since Th2 cells are not the only source of Th2 cytokines in the lungs in allergic asthma, AHR and lung secretion of IL-4 and IL-5 induced by OVA was increased in both A/J and BALB/c. The role of Th17 cells in allergic asthma is less clear than Th2 cells; experimental models have shown IL-17 to exert both anti-inflammatory and proneutrophilic inflammatory effects, indicating distinct activities in sensitization and challenge phases [36]. Here, A/J naïve mice presented reduced frequency of Th17 cells in the lungs, compared to C57BL/6, but IL-17 lung production was not different in the 3 mouse strains after the antigenic challenge.

The role of Foxp3^+^ Tregs in asthmatic patients is also a matter of controversy; it has been shown that adult patients with severe symptoms present low amounts of Tregs in the airways and blood [13, 14], while other studies show the number of lung regulatory lymphocytes to be increased in severe asthma compared with mild asthmatics and healthy subjects [16], especially following inhaled allergen challenge [17]. In mice, inhalation of OVA leads to tolerance mediated by Foxp3^+^ Tregs [37], unless it is administered with an adjuvant combined with priming through subcutaneous or intraperitoneal routes. Different models of experimental asthma using OVA combined with an adjuvant can induce lung inflammation and AHR, mimicking the main features of the disease. Data on the frequency of Foxp3 Treg cells in murine experimental asthma are not easily compared, since they either lack nonallergic control mice and Foxp3 intracellular staining on CD4^+^CD25^+^ cells or use only one mouse strain [23, 38, 39]. Nonetheless, the immunosuppressive role of these cells has been confirmed, since transfer of Tregs can suppress the main features of asthma in mice, through secretion of IL-10 and TGF-*β* [26]. In our study, C57BL/6 mice did not develop lung inflammation or AHR with the allergenic protocol used, which could be a consequence of the higher frequency of Foxp3^+^ Tregs in their airways and of the higher expansion of these cells upon an OVA *in vitro* booster, compared to cells from the other strains. On the other hand, OVA-challenged A/J mice presented robust lung inflammation and AHR, but Foxp3^+^ Treg cells obtained from their lung draining lymph nodes did not expand in the presence of OVA *in vitro* and had limited expansion when OVA was used with TGF-*β*, an inducer of Treg cell generation and expansion [25, 40]. Nonallergic *in vitro* stimulus of naïve lymph node cells with anti-CD3 and anti-CD28 in the presence of TGF-*β* led to an increased expansion of the CD4^+^CD25^+^Foxp3^+^ cell population in cells obtained from all mice, but this increase was significantly higher in cells from BALB/c mice, compared to A/J and C57BL/6. Interestingly, when cells are obtained from the lung draining lymph nodes of OVA-challenged mice, BALB/c presented higher frequency of the CD4^+^CD25^+^Foxp3^+^ cell population, but upon an OVA antigenic *in vitro* booster, there was a clear increase in the frequency of CD4^+^CD25^+^Foxp3^+^ cell population in cells from C57BL/6 mice. An *in vitro* study using human CD4 T cells showed enhancement of Foxp3 expression induced by 1*α*25VitD3 that can be impaired by IL-10 [41]. It is possible, therefore, that the difference in the frequency of CD4^+^CD25^+^Foxp3^+^ cells among the OVA-challenged strains is a consequence of the levels of IL-10 secreted, since A/J mice present high levels of IL-10 and smaller frequency of Foxp3^+^ Tregs.

In our study, naïve A/J mice presented higher frequency of CD4^+^IL-10^+^ cells in the lung parenchyma and airways, when compared to BALB/c and C57BL/6 naïve animals. OVA-sensitized and saline-challenged A/J mice also presented higher IL-10 production in the lungs, compared to the other strains. OVA lung challenge further increased lung IL-10 production in both A/J and BALB/c mice but not in C57BL/6. Allergenic *in vitro* booster of A/J mouse mediastinal lymph node cells obtained from OVA-challenged mice led to a significant increase in percentage of CD4^+^IL-10^+^ cells, showing these cells are responsive to OVA. Additionally, the increase in the frequency of CD4^+^IL-10^+^ cells correlated with enhanced IL-10 production *in vitro*, suggesting these cells are responsible, at least in part, for the IL-10 increased production in A/J mouse. Domínguez-Punaro and cols assessed the response to *Streptococcus suis* in different mouse strains and demonstrated that A/J mice had increased susceptibility to the disease due to an increased inflammatory response, when compared to C57BL/6 mice [42]. In their model, enhanced production of IL-10 in C57BL/6 infected mice was associated to their higher survival. They also provided evidence that the IL-10 treatment of A/J infected mice had protective effect, pointing out that A/J mice are responsive to IL-10, and this cytokine may have a role in the susceptibility to this inflammatory condition.

Although there are still no data showing unresponsiveness to IL-10 in A/J mice, we cannot rule out possible disfunctions on the IL-10, IL-10 receptor (IL-10R), or the downstream signaling pathway. Polymorphisms of IL-10, IL-10R, and STAT3, a Janus kinase (JAK)–signal transducer and activator of transcription, activated downstream of IL-10R were found to be associated to asthma and higher levels of IgE, without interfering with IL-10 levels [43, 44]. Nonetheless, IL-10 production is sustained by IL-10R in mouse and human Tregs [45], suggesting that CD4^+^IL10^+^ cells are responsive to IL-10 in A/J mice. Moreover, IL-10 displays pleiotropic effects in key inflammatory cells of asthma; for instance, it was described to have distinct roles in IgE production, either inhibiting IgE switching or potentiating IgE production on IgE isotype switched cells [46, 47]. A dual role of IL-10 on the production of IL-5 and IL-13 was also described; interestingly, upregulation of Th2 cytokines was found when overexpression of IL-10 was induced during the late immune response [48]. If Th2 cytokine and IgE increased production on allergenic-challenged A/J mice [32, 33] is linked to their higher levels of the lung secreted, IL-10 is still a matter that needs further investigation.

In conclusion, the present study suggests that the observed differences in the frequencies of allergen-responsive Foxp3 or IL-10 CD4 T cells are associated with the distinct susceptibility of A/J, BALB/c, and C57BL/6 mice to experimental asthma. These characteristics are crucial and should be considered upon choosing a mouse strain to study difficulty-to-treat asthma and to screen novel antiasthma candidate compounds.

## Data Availability

Data available on request from the authors.

## Additional Points

*Key Messages*. Foxp3 or IL-10 Tregs have distinct frequencies in different mouse strains. Differences in allergen-responsive Treg frequencies are associated with distinct susceptibility to experimental asthma. These characteristics should be considered upon choosing a mouse strain to study asthma and antiasthmatics.
